# Heartbeat optical coherence elastography: corneal biomechanics *in vivo*

**DOI:** 10.1117/1.JBO.26.2.020502

**Published:** 2021-02-23

**Authors:** Achuth Nair, Manmohan Singh, Salavat Aglyamov, Kirill V. Larin

**Affiliations:** a University of Houston, Department of Biomedical Engineering, Houston, Texas, United States; b University of Houston, Department of Mechanical Engineering, Houston, Texas, United States

**Keywords:** cornea, tissue biomechanics, optical coherence elastography, optical coherence tomography, *in vivo*, ocular pulse

## Abstract

**Significance:** Mechanical assessment of the cornea can provide important structural and functional information regarding its health. Current clinically available tools are limited in their efficacy at measuring corneal mechanical properties. Elastography allows for the direct estimation of mechanical properties of tissues *in vivo* but is generally performed using external excitation force.

**Aim:** To show that heartbeat optical coherence elastography (Hb-OCE) can be used to assess the mechanical properties of the cornea *in vivo.*

**Approach:** Hb-OCE was utilized to detect Hb-induced deformations in the rabbit cornea *in vivo* without the need for external excitation. Furthermore, we demonstrate how this technique can distinguish corneal stiffness between untreated (UT) and crosslinked (CXL) tissue.

**Results:** Our results demonstrate that stiffness changes in the cornea can be detected using only the Hb-induced deformations in the cornea. Additionally, we demonstrate a statistically significant difference in strain between the UT and CXL corneas.

**Conclusions:** Hb-OCE may be an effective tool for assessing the mechanical properties of the cornea *in vivo* without the need for external excitation. This tool may be effective for clinical assessment of corneal mechanical properties because it only requires optical coherence tomography imaging and data processing.

## 1 Introduction

The cornea is an essential component of vision, and various pathologies, such as keratoconus and corneal ectasia, can affect its structural integrity and biomechanical properties.^1^^,^^2^ One emerging method for assessing these biomechanical changes in corneal tissue is utilizing noncontact tonometry, such as with the CorVis ST (OCULUS Optikgerate GmbH, Germany) and ORA (Reichert Technologies). While these tools are useful in the clinic, separating the effects of stiffness, intraocular pressure (IOP), and corneal geometry with these devices is a complex problem that is still under investigation.^3^^–^^7^

Elastography is an established technique for directly assessing the mechanical properties of tissues.^8^^–^^10^ Ultrasound elastography techniques have been very useful for evaluating the mechanical properties of tissues *in vivo* and *ex vivo*, including the cornea.^11^^,^^12^ Optical elastography techniques, including Brillouin microscopy and optical coherence elastography (OCE) have also been used to investigate corneal mechanical properties *in vivo*.^8^^,^^13^^–^^15^ Brillouin microscopy is an exciting tool for assessing tissue mechanical properties. However, spatial mapping of the cornea is limited due to relatively long imaging times, and evaluating the relationship between the Brillouin modulus and Young’s modulus is still under investigation.^15^^–^^17^ OCE of the cornea *in vivo* has been performed using multiple excitation techniques.^18^^–^^22^ While these methods were effective for assessing the mechanical properties of the cornea, there is increasing interest in measuring the biomechanical properties of tissues without any external excitation.^23^^–^^25^ In this form of elastography, tissue stiffness is assessed by physiologically induced displacements. Without an external excitation source, mechanical measurements can be performed with limited additional components; clinicians can assess tissue mechanical properties using only the essential imaging technology. This is particularly advantageous in the field of ophthalmology, where optical coherence tomography (OCT) is ubiquitous. Nguyen et al.^26^ demonstrated passive elastography in ocular tissue for the first time in the *in vivo* rat eye. Kling^27^ and Kling et al.^28^ used another passive technique to measure the mechanical properties of the cornea using ambient introcular pressure modulation.

In our previous work, we introduced the Hb-OCE method in an *ex vivo* porcine model with a simulated ocular pulse.^24^ This technique bears some similarity to the work done by Nguyen et al.^26^ due to the passive nature of excitation. However, where Nguyen assessed mechanical response to diffuse shear wave propagation induced by natural motions, the technique proposed in this work directly assesses the tissue response to the Hb-induced ocular pulse, similar to measurements performed using ultrasonic techniques.^23^^,^^29^ In this letter, we demonstrate that Hb-OCE can measure changes in tissue stiffness induced by crosslinking (CXL) *in vivo* in a rabbit model.

## 2 Methods

In these pilot studies, Hb-OCE measurements were acquired in two Dutch-Belted rabbits. One rabbit was untreated (UT), while the other was treated with the standard collagen crosslinking treatment to stiffen the cornea. Animals were anesthetized with an initial intramuscular dose of 40-mg/kg ketamine and 5-mg/kg xylazine by a veterinarian. IOP was measured using a handheld rebound tonometer (Tonovet, Icare Finland Oy, Vantaa, Finland). The animals were placed in a lateral recumbent position in a custom-made rest fitted with a heating pad to ensure the rabbit remained at a comfortable temperature. Vital signs were monitored by a veterinarian, and a maintenance dose of 20  mg/kg ketamine was injected intramuscularly as needed. The rabbits were placed on a three-axis stage for easy orientation of their eye under the OCT sample arm. While anesthetized, the rabbits were unable to blink, so corneal hydration was maintained with 1× phosphate-buffered-saline (PBS) applied at regular intervals. All animal procedures were approved by the University of Houston Institutional Animal Care and Use Committee.

A spectral domain OCT system with 840-nm central wavelength, 49-nm bandwidth, 6-μm axial resolution, 8-μm lateral resolution, and 62.5-kHz line rate was used for OCT imaging of the rabbit cornea. B-M-mode scans with A-line size of 500 over a 4-mm region at the apex of the cornea were acquired at a 100-Hz frame rate.

**Fig. 1:**
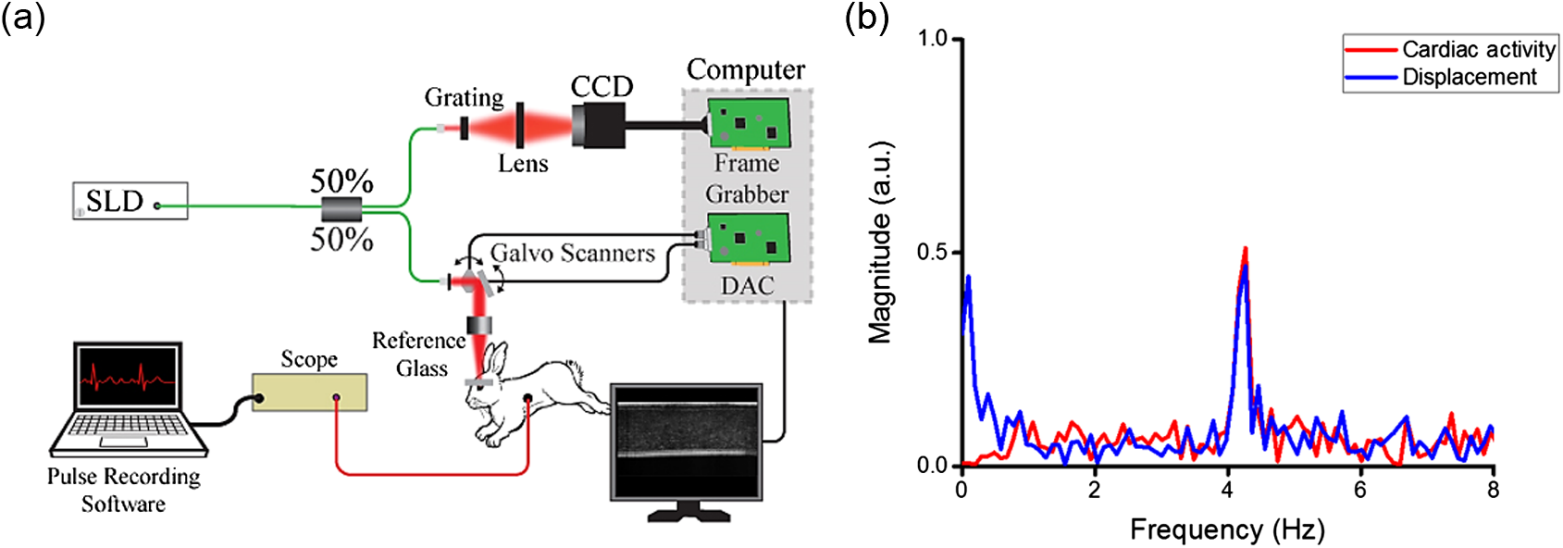
(a) OCE system schematic diagram. Corneal displacements in response to Hb were detected using an SD-OCT system. Hb was monitored using a pressure transducer placed over the chest. (b) Cardiac activity measured by chest-mounted pressure transducer, and corneal displacement measured by Hb-OCE. Frequency of the displacement detected coincides with the heart rate.

To minimize eye motion due to saccades and breathing, a glass window was placed in the OCT sample arm in contact with the cornea. The window also allowed for imaging in the common path configuration,^30^^,^^31^ and improved the displacement stability to <1  nm at a signal-to-noise ratio of 108 dB. A pressure transducer (PowerLab, ADInstruments, Dunedin, New Zealand) was placed on the chest of the rabbits for heart rate monitoring and recorded throughout the imaging procedure. The Hb data was later coregistered temporally with the OCE data. Figure 1 shows a schematic diagram of the OCE system.

Measurements were obtained from the areas flattened by the glass window over the ∼4  mm imaging region. CXL followed the standard Dresden protocol. Briefly, the epithelium was removed, and a solution of 0.1% riboflavin in 20% dextran was applied over a 30 min period at 5-min intervals on the surface of the stromal surface. The application of the solution was then repeated for another 30 min with UV irradiation.^32^

OCT images were acquired over several seconds to capture corneal displacement over many cardiac cycles. Motion detection in the cornea and strain calculation was determined using an algorithm detailed in our previous work.^24^ Briefly, motion between successive frames was detected using a complex conjugate method.^33^^,^^34^ Noise removal was performed using vector averaging over a 6×6 kernel size.^35^ Displacement was calculated using a two-dimensional (2D) spatial unwrapping step for each frame.^36^ A low-pass filter with a 12-Hz cut-off frequency was used to eliminate high-frequency noise over the imaging period at each spatial position. The strain was measured using a least-squares regression algorithm at each lateral position (i.e., for each A-line) of every frame, using a kernel size of 150  μm.^37^ Strain data were masked by the intensity of the structural image to eliminate motion from regions not corresponding to the cornea. The strains were then averaged for each frame for analysis.

## 3 Results

Figure 1 shows normalized frequency spectra of the displacement in the cornea of a UT rabbit measured by Hb-OCE and the Hb measured from the pressure transducer on the rabbit chest. The frequency of the displacement detected coincides with the heart rate. The animal heart rate was 4.3 Hz, which corresponded to ∼258  bpm and was consistent with vital sign measurements performed by a veterinarian. This shows that the source of deformation for Hb-OCE is indeed the result of cardiac activity.

**Fig. 2:**
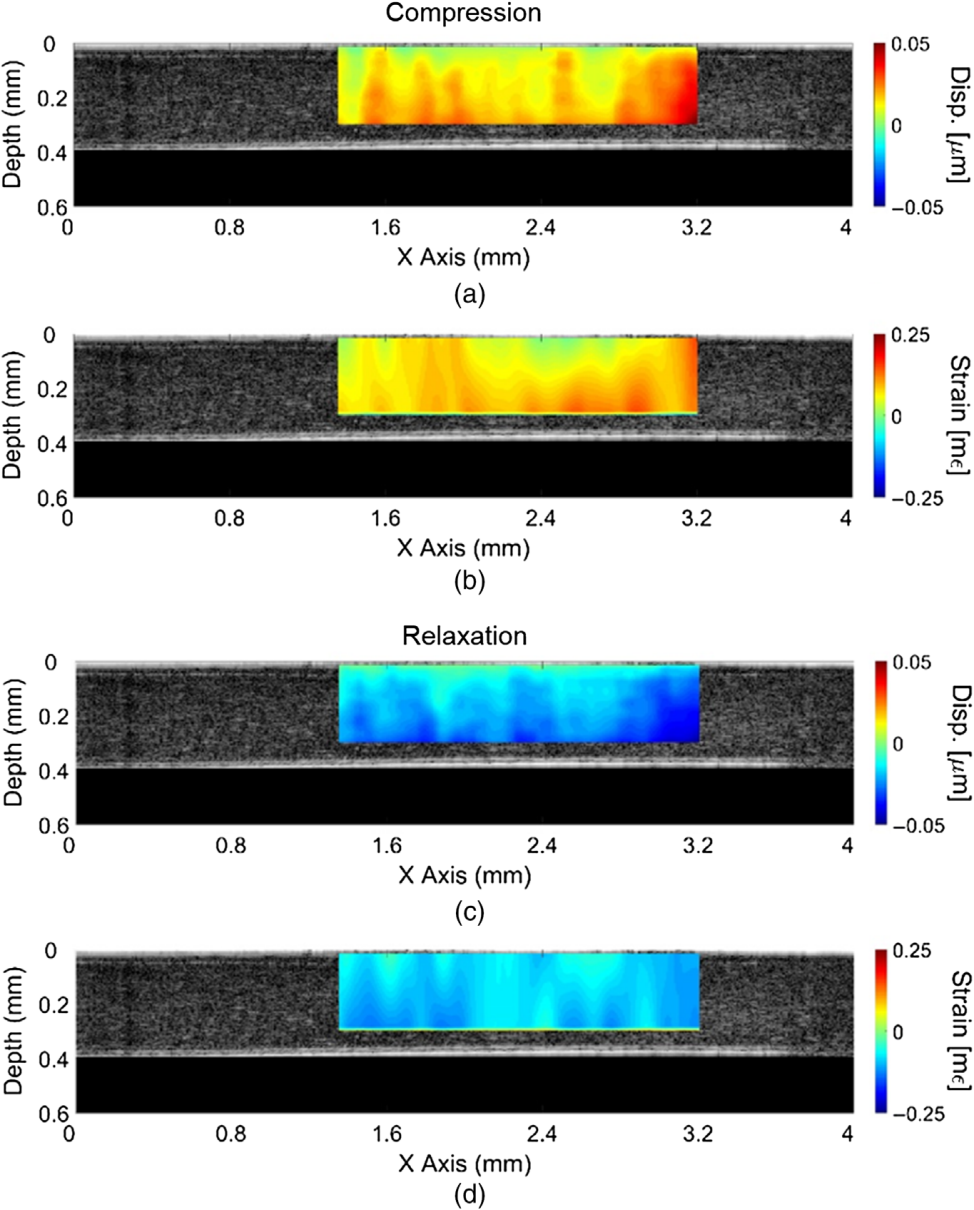
Spatially distributed displacement and strain for corneal during ocular pulsation. (a) and (b) correspond to displacement and strain, respectively, for corneal compression. (c) and (d) correspond to displacement and strain, respectively, for corneal relaxation (decompression).

Figure 2 shows the spatial distribution of ocular pulse-induced displacement and strain in the UT cornea. The displacement and strain were mapped at the instantaneous (between two frames) moment of peak corneal compression and decompression, respectively. Compressive strain is reported as a positive number, and expansion is reported as negative to maintain consistency with our previous work. The region of interest shown corresponds to the area of the cornea with best contact with the reference glass. The cornea exhibited distinct strain with compression and relaxation (decompression) that corresponded to the Hb. Heterogeneity in the strain maps may be due to nonuniform contact between sample and the reference glass or friction.^38^ While the corneas were regularly hydrated with PBS, different solutions may provide better lubrication between the cornea and glass.

**Fig. 3:**
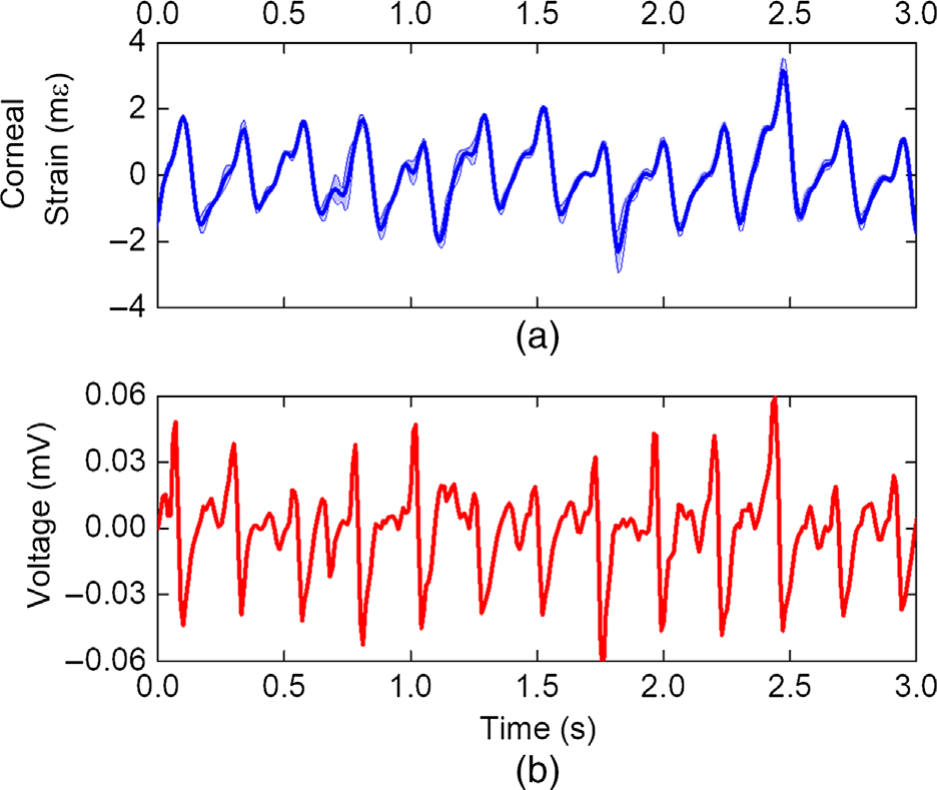
(a) Corneal strain over time measured *in vivo* in the UT cornea. (b) Cardiac activity monitored by chest mounted pressure transducer.

The strain in the cornea was calculated for every pair of successive OCT B-frames, and the cumulative sum showed the strain relative to the initial frame. The mean and standard deviation of the strain was then calculated for each strain frame. The average strain was insensitive to the heterogeneity shown in Fig. 2. Average strain and Hb measured by the pressure transducer are shown in Fig. 3, where the measured strain dynamics mirror the heart rate. The strain shows a double peak at the rising edge, where this behavior is likely related to the Hb as a similar behavior is seen from the cardiac measurement.

**Fig. 4:**
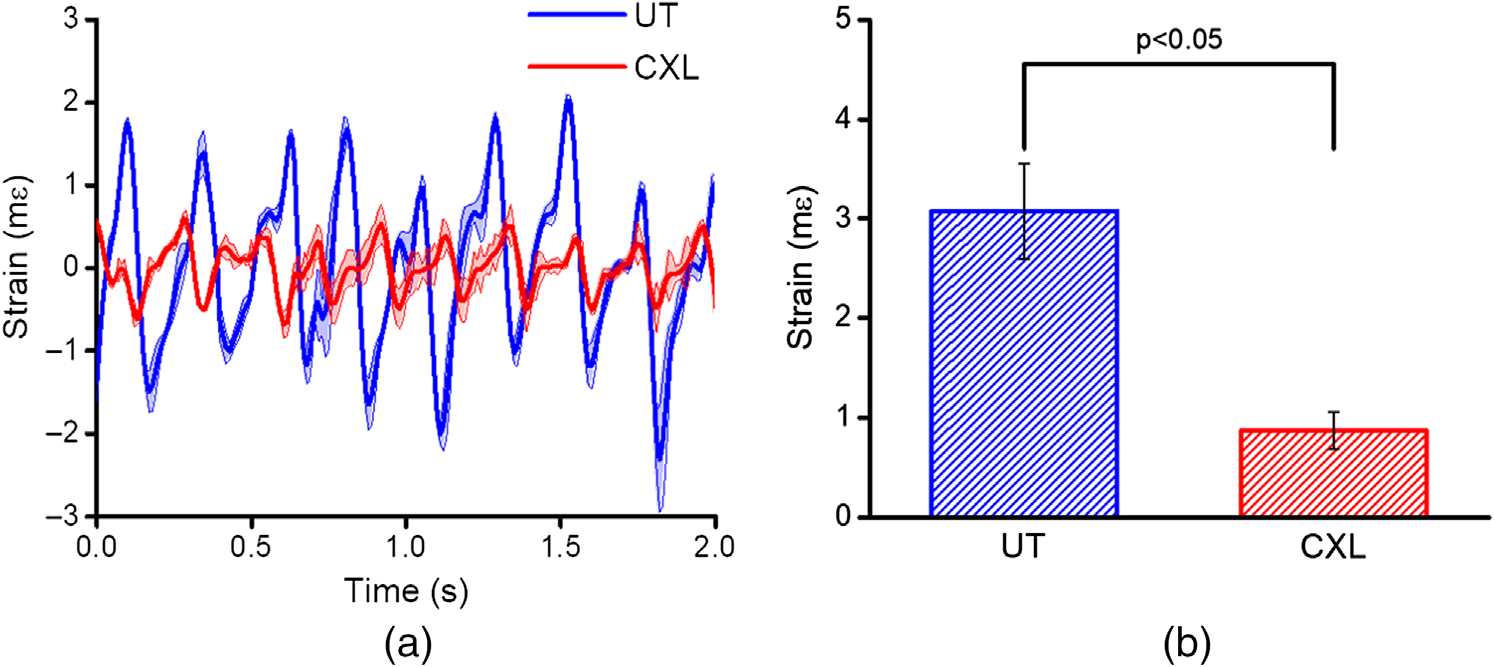
(a) Strain over time for UT and CXL cornea. (b) Mean and standard deviation of strain for the UT and CXL cornea over 12 pulses.

While data shown in Fig. 3 show the feasibility of Hb-OCE *in vivo*, we have also performed controlled changes in tissue stiffness by CXL to assess the ability of this method to detect changes in stiffness. Figure 4 shows the strain detected in the cornea of a rabbit before (UT) and after CXL. Both eyes had a mean IOP of 10 mmHg detected by the rebound tonometer. The peak-to-peak amplitude of strain at individual ocular pulses for the UT and CXL cornea was calculated. The mean and standard deviation for the strain values of 12 consecutive pulses is shown in Fig. 4(b) for the UT and CXL corneas. The UT cornea had a mean strain of 3.07±0.48  mε, and the CXL cornea had a mean strain of 0.87±0.19  mε. A two-sample t-test showed a statistically significant difference (p<0.05) between the two sample types.

## 4 Discussion and Conclusion

Traditionally, *in vivo* assessment of corneal biomechanics has been performed using dynamic wave propagation and quasistatic elastography.^18^^,^^20^^,^^39^ These tools can provide important mechanical information, but often require complex analysis or additional tools to properly calculate elasticity.^40^^,^^41^ Compared to similar methods such as compression-based OCE, the strain we see here is much lower, which is due to the much smaller displacement induced by the ocular pulse as compared to compression by a mechanical actuator. The results of this work suggest that Hb-OCE is still effective for *in vivo* assessment of corneal mechanical properties. While the current study utilized a healthy animal models our results suggests that this technology may be effective in measuring corneal stiffness in a disease model. Using Hb-OCE, clinical assessment of mechanical properties of the cornea using only an OCT imaging system, a ubiquitous tool in ophthalmology and optometry clinics, is feasible.

While the results of this work do provide an exciting development for this technique, there are some limitations. Hb-OCE was initially debuted as a noncontact tool for the assessment of elasticity.^24^ However, in its current configuration, it requires an optical window placed on the corneal surface to reduce out-of-plane motion, minimize bulk motion, and increase stability. This compression technique may also affect IOP. Though compression with a glass window does have limitations in terms of comfort, it has been utilized in the clinic.^18^^,^^19^ Additionally, though common-path OCT does require an additional piece of hardware, it is a minor alteration to an OCT system. Comparison between dual-arm and common path OCE are beyond the scope of this work but have been explored previously.^30^^,^^34^ The proposed technique used a reference glass to restrict out-of-plane motion, and the 100-Hz frame rate was useful for capturing small strains with minimal speckle decorrelation. Repeatability could be improved with faster imaging speeds and advanced motion correction algorithms, which are in development. Additionally, there may be lateral heterogeneity in the cornea since the corneal thickness is not uniform from the apex to the limbus. Our future work involves measuring the ocular-pulse-induced strain without contact to measure the stiffness of the cornea over a larger region.

Our results clearly show that mechanical properties can be measured with a glass reference window, but we believe that the Hb-OCE can be performed without any contact, as it was performed *ex vivo* in our previous work. In that study, the cornea exhibited a displacement while the eye was in a holder, like the ocular orbit *in vivo*. For a noncontact implementation of Hb-OCE *in vivo*, motion compensation can be accomplished by utilizing faster imaging speeds that would allow volumetric speckle tracking and/or minimize interframe decorrelation and, thus, effective correction for motion artifacts.

In these preliminary studies, we demonstrated a qualitative assessment of corneal stiffness. This type of analysis has limitations and may be dependent on ocular pulse amplitude and baseline IOP. However, there are tools that can measure these parameters clinically, so the strain could be easily converted to quantitative elasticity (e.g., Young’s modulus). Another way to estimate Young’s modulus involves using a stress sensor as in quantitative microelastography; adding such a sensor is an area of future exploration. Truly quantitative measurement of the stiffness, such as Young’s modulus, might also require analytical models that also account for stress on the cornea and the aforementioned IOP.^42^ Quantitative assessments may also be useful for assessing corneal stiffness in patients with ocular and cardiovascular pathologies that may affect blood flow. These conditions may change ocular pulse shape and subsequent strain, which is an important area of further investigation.^43^
